# Cholestatic and autoimmune liver diseases, bile duct injury, oxidative stress, and therapeutic strategies

**DOI:** 10.3389/fphys.2026.1788973

**Published:** 2026-05-13

**Authors:** Dominika Stygar, Bartosz Bogielski, Katarzyna Michalczyk, Bronisława Skrzep-Poloczek, Alicja Suchocka, Kamil Nikiel, Kazimierz Kukla, Rafał Wasek, Jakub Staniszewski, Maciej Matyja, Michał Kukla

**Affiliations:** 1 Department of Physiology, Faculty of Medical Sciences in Zabrze, Medical University of Silesia, Zabrze, Poland; 2 Bielsko-Biała Branch, Medical University of Silesia, Bielsko-Biała, Poland; 3 Second Department of General Surgery, Jagiellonian University Medical College, Kraków, Poland; 4 Department of Internal Medicine and Geriatrics, Jagiellonian University Medical College, Kraków, Poland; 5 Department of Endoscopy, University Hospital in Kraków, Kraków, Poland

**Keywords:** bile acid metabolism, cholangiocytes, cholestasis, intrahepatic cholestasis of pregnancy (ICP), primary biliary cholangitis (PBC), primary sclerosing cholangitis (PSC), reactive oxygen species (ROS)

## Abstract

Cholestatic and autoimmune liver diseases, including primary sclerosing cholangitis (PSC), primary biliary cholangitis (PBC), and intrahepatic cholestasis of pregnancy (ICP), represent a heterogeneous group of conditions characterized by impaired bile flow, bile duct injury, and progressive hepatobiliary dysfunction. Oxidative stress has emerged as a critical factor linking bile acid toxicity to inflammation, fibrosis, and loss of biliary epithelium. This review synthesizes current evidence on the pathophysiological interplay between bile duct injury and oxidative stress in PSC, PBC, and ICP, with an emphasis on the role of redox imbalance in disease progression and therapeutic response. Across all three diseases, bile acid accumulation and mitochondrial dysfunction generate reactive oxygen species (ROS) that induce lipid peroxidation, DNA damage, and apoptosis in hepatocytes and cholangiocytes. Biomarkers, including malondialdehyde (MDA), 8-hydroxydeoxyguanosine (8-OHdG), and derivatives of reactive oxygen metabolites, correlate with disease severity and prognosis. Current therapeutic strategies target both bile acid metabolism and oxidative stress. Ursodeoxycholic acid (UDCA) enhances glutathione synthesis and reduces lipid peroxidation, while newer agents, such as obeticholic acid (OCA) and fibrates, act via farnesoid X receptor (FXR) and peroxisome proliferator-activated receptor (PPAR) signaling to modulate bile acid homeostasis and redox balance. However, clinical outcomes remain variable, and long-term benefits are not fully established.

## 1 Introduction

Cholestatic liver diseases represent a spectrum of disorders characterized by the impaired bile flow, that leads to the accumulation of bile acids and other toxic constituents within the liver. This disruption not only causes direct hepatocellular injury, but also initiates a complex cascade of inflammatory and fibrotic processes, ultimately progressing to cirrhosis and end-stage liver disease if left unchecked. Among these, primary sclerosing cholangitis (PSC), primary biliary cholangitis (PBC), and intrahepatic cholestasis of pregnancy (ICP) stand as prime examples where cholestasis is intertwined with autoimmune and hormonally-mediated pathogenic mechanisms. The review aims to synthesize current knowledge on the intricate interplay between bile duct injury, oxidative stress, and emerging therapeutic strategies in these three distinct, yet pathophysiologically linked conditions (Hirschfield et al., 2010; Marchioni Beery et al., 2014; Hilscher et al., 2020; Zeng et al., 2023).

The hallmark of PBC and PSC is an immune-mediated attack on the biliary epithelium. Primary biliary cholangitis primarily targets the small intrahepatic bile ducts, characterized by the presence of highly specific anti-mitochondrial antibodies (AMAs), often against the E2 subunit of the pyruvate dehydrogenase complex (PDC-E2) (Nakamura et al., 2005; Hirschfield and Gershwin, 2013) In contrast, PSC affects both intra- and extrahepatic bile ducts of larger caliber, leading to concentric periductal fibrosis, structuring, and the classic “beaded” appearance on cholangiography, often in association with inflammatory bowel disease (Karlsen et al., 2017). While ICP is typically a self-limiting condition occurring in the third trimester of pregnancy, it shares the fundamental pathophysiological feature: the toxic retention of bile acids, particularly hydrophobic acids like deoxycholic acid and chenodeoxycholic acid (Williamson and Geenes, 2014).

A critical consequence of bile acid accumulation is the induction of severe oxidative stress. Hydrophobic bile acids are potent inducers of reactive oxygen species (ROS) generation, which overwhelms the hepatic antioxidant defense system, comprising enzymes such as superoxide dismutase (SOD), catalase (CAT), and glutathione peroxidase (GPx) (Sokol et al., 2001; Shearn et al., 2018; Wang MQ. et al., 2024). This redox imbalance leads to lipid peroxidation, evidenced by increased levels of malondialdehyde (MDA), protein damage, and DNA injury, that further propagate inflammation and apoptosis of hepatocytes and cholangiocytes (Liu et al., 2024; Zheng et al., 2024). Cholestasis promotes the accumulation of ROS and reactive aldehydes around periportal areas, while dysregulation of CAT, SOD, and glutathione-related enzymes further amplifies cellular injury (Shearn et al., 2018). In PBC, markers of systemic oxidative stress, including total antioxidant capacity (TAC) and total oxidative stress (TOS), correlate with disease progression, highlighting a potential prognostic feature of the oxidative imbalance (Dallio et al., 2024b). These findings are supported by molecular studies demonstrating impaired redox responses in both PSC and PBC (Petersen et al., 2018; Ortiz et al., 2023).

Therapeutic paradigms are evolving beyond mere symptom management to target these core pathogenic pathways. Ursodeoxycholic acid (UDCA), a hydrophilic bile acid, remains the first-line therapy in PBC and is used in ICP for its choleretic and cytoprotective effects, partly attributed to its ability to mitigate oxidative stress (Paumgartner and Beuers, 2002). For UDCA-non-responsive PBC patients, obeticholic acid (OCA), a potent farnesoid X receptor (FXR) agonist, offers a newer alternative that also modulates bile acid metabolism and exhibits anti-inflammatory and antioxidant properties (Nevens et al., 2016). Furthermore, fibrates, traditionally used for dyslipidemia, have emerged as promising adjuncts in PBC treatment. Their agonism on peroxisome proliferator-activated receptors (PPARs), notably PPAR-α and PPAR-γ, not only improves lipid metabolism, but also exerts direct anti-cholestatic, anti-inflammatory, and protective effects on cholangiocytes (Corpechot et al., 2018).

This review will provide a comprehensive overview of PSC, PBC, and ICP, delving into their autoimmune and cholestatic nature, the mechanisms of bile duct destruction, and the central role of oxidative stress in their pathophysiology. We will detail the enzymatic and immunological markers of disease and critically evaluate current and novel therapeutic strategies aimed at counteracting bile acid toxicity and oxidative damage to improve patient outcomes.

## 2 Materials and methods

The content of the review was systematically gathered by searching the PubMed, Scopus, and Google Scholar databases to collect and analyze the available literature on cholestatic and autoimmune liver diseases, with a specific focus on bile duct injury, oxidative stress, and therapeutic strategies. A comprehensive search strategy was employed using a combination of keywords and phrases, including but not limited to: “oxidative stress markers,” “prognosis,” “total antioxidant capacity,” “total oxidative stress,” “catalase,” “superoxide dismutase (SOD),” “glutathione peroxidase,” “glutathione reductase,” “inflammation,” “antioxidative defense system,” “anti-catalase antibodies,” “reactive oxygen species (ROS),” “bile acid metabolism,” “cholestatic disease,” “lipid peroxidation,” “malondialdehyde (MDA),” “heat shock proteins 70 (Hsp70),” “cholangiocytes,” “cholestasis,” “thioredoxins,” “hepatocellular response,” “human hepatocytes,” “primary biliary cholangitis (PBC),” “alpha-enolase autoantibodies,” and “reactive aldehydes.” These terms were applied individually or in multiple combinations to ensure both breadth and relevance in covering the scope of the topic.

The search was restricted to original research articles and review papers reporting empirical data in both animal models and human subjects. The time frame for inclusion was set from January 1990 to February 2026, providing a historical perspective for analyzing the incorporated more recent findings. To maintain clarity and consistency, only articles published in English were considered, with non-English studies excluded to avoid potential discrepancies from translation.

The initial search results underwent a two-stage screening process. First, titles and abstracts were examined to determine relevance to the objectives of this review, excluding the irrelevant studies or those not directly addressing cholestatic or autoimmune liver diseases, bile duct pathology, oxidative stress, or therapeutic interventions. In the second stage, full-text articles were assessed in detail, and further exclusions were made in cases of insufficient data, lack of focus on oxidative stress or therapeutic strategies, or methodological limitations that could compromise reliability.

Ultimately, the selected studies were critically appraised and synthesized to provide an integrative overview of the interplay between bile duct injury, oxidative stress, and therapeutic approaches in cholestatic and autoimmune liver diseases.

## 3 Primary sclerosing cholangitis

Primary sclerosing cholangitis is a chronic, progressive fibro-inflammatory cholangiopathy that affects the intra- and/or extrahepatic bile ducts. It is strongly associated with inflammatory bowel disease (IBD), and carries an increased risk of cholangiocarcinoma; many patients ultimately progress to biliary structuring, cholestasis, and liver failure. Diagnosis rests on a cholestatic biochemical profile and characteristic cholangiographic abnormalities (with small-duct variants showing normal cholangiography but PSC-type histology) (Bowlus et al., 2023).

Current evidence indicates that bile-duct injury in PSC arises from an interaction of genetic susceptibility, disordered gut–liver crosstalk, and maladaptive biliary epithelial (cholangiocyte) responses. Multiple studies demonstrate intestinal dysbiosis specific to PSC, distinct from “IBD alone,” supporting a role for microbial products and altered bile-acid metabolism in driving periductal inflammation and fibrosis (Kevans et al., 2016; Sabino et al., 2016).

At the duct wall, cholangiocyte senescence (an arrested, pro-inflammatory state) is prominent across disease stages and correlates with histologic severity and outcomes; senescent cholangiocytes secrete cytokines, chemokines, and profibrotic factors that perpetuate periductal “onion-skin” fibrosis (Cazzagon et al., 2021). Experimental and translational work further shows that biliary constituents can trigger cholangiocyte injury and senescence (e.g., via N-Ras signaling), linking cholestasis to progressive duct damage (Tabibian et al., 2014). Evidence shows that its progression is closely associated with oxidative stress and impaired antioxidant defenses (Petersen et al., 2018; Shearn et al., 2018; Shearn et al., 2019; Oyama et al., 2023). Guideline statements synthesize these data into a unifying model: in genetically predisposed hosts, a microbiota-modulated immune response targets the biliary tree; injured cholangiocytes adopt a senescence-associated secretory phenotype, amplify immune activation, and recruit myofibroblasts, leading to concentric periductal fibrosis and fixed biliary strictures. This pathobiology explains the typical cholangiographic “beading,” frequent IBD association, and the increased cancer risk in PSC (European Association for the Study of the Liver, 2022).

## 4 Primary biliary cholangitis

Primary biliary cholangitis is a chronic autoimmune liver disease characterized by a progressive, non-suppurative inflammatory process that leads to T lymphocyte–mediated destruction and necrosis of small interlobular and septal bile ducts. This injury is accompanied by infiltration and proliferation of immune cells into the hepatic parenchyma, ultimately resulting in cholestasis. Over time, fibrosis develops through the activation of myofibroblastic cells in the hepatic mesenchyme, progressing to cirrhosis and loss of liver function. Importantly, bile ducts lack regenerative capacity, which contributes to the irreversible nature of the disease (Kumagi and Heathcote, 2008; Lindor et al., 2009a; Zhao et al., 2023).

Diagnosis of PBC requires the fulfillment of at least two of the following three criteria: (1) biochemical evidence of cholestasis, particularly elevated alkaline phosphatase levels; (2) the presence of disease-specific autoantibodies, most commonly anti-mitochondrial antibodies (AMA), or alternatively Sp100 and GP210; (3) histological findings consistent with non-suppurative destructive cholangitis and interlobular bile duct loss (Barba Bernal et al., 2023).

The disease predominantly affects middle-aged women (Barba Bernal et al., 2023) and is thought to arise in genetically predisposed individuals exposed to environmental triggers such as viral or bacterial infections, xenobiotics, or immune dysregulation (Reshetnyak, 2015). The typical histopathological picture is defined by ductopenia—absence of interlobular bile ducts in more than 50% of portal tracts (Bhandari et al., 2011)—along with portal infiltration by T and B lymphocytes, macrophages, eosinophils, and natural killer cells (Nguyen et al., 2010). AMAs represent a hallmark of autoimmune etiology, targeting mitochondrial antigens of the M2 family, including the E2 subunit of the pyruvate dehydrogenase complex (PDC-E2), 2-oxoglutarate dehydrogenase complex (OGDC), and branched-chain oxoacid dehydrogenase (BCOADC). Recognition of these antigens triggers both cellular and humoral immune responses, leading to progressive bile duct inflammation and destruction (Kumagi and Heathcote, 2008; Zhao et al., 2024).

Oxidative stress has emerged as a key driver of disease progression in PBC. Normally, hepatocytes are protected from the damaging effects of ROS through robust antioxidant systems; however, excessive ROS accumulation combined with impaired antioxidant defenses promotes oxidative injury. Experimental studies in rats with bile duct ligation (BDL) demonstrated elevated lipid peroxidation in hepatic mitochondria, reflected by increased MDA and 4-hydroxynonenal (HNE) levels, correlating with neutrophil and monocyte infiltration (Copple et al., 2010). Additional findings in both animals and humans indicate decreased levels of vitamin E and reduced activity of glutathione peroxidase, glutathione transferase, and catalase, further disrupting redox homeostasis. Accumulated bile acids themselves exacerbate ROS generation and promote biliary epithelial cell (BEC) senescence, amplifying bile duct injury (Onofrio et al., 2019).

At the cellular level, BECs in PBC exhibit dysregulated cell cycle control, undergoing premature senescence and apoptosis. Overexpression of p21^WAF1/Cip1 and p16^INK4 has been implicated as a potential pathway underlying this process (Sasaki et al., 2008). In a study by (Sasaki et al., 2008), liver biopsy samples from patients with early-stage PBC revealed strong expression of oxidative stress markers—including phosphorylated ATM (p-ATM), 8-hydroxydeoxyguanosine (8-OHdG), and p21^WAF1/Cip1—within the nuclei of damaged bile ducts, bile ductules, and periportal hepatocytes. Co-localization of these markers suggested ATM pathway activation during oxidative stress, correlating with apoptosis of BECs and impaired regenerative capacity (Sasaki et al., 2008). Furthermore, perinuclear infiltration of myeloperoxidase-positive (MPO) inflammatory cells reinforces the role of oxidative stress–mediated inflammation in disease progression (Dallio et al., 2024a).

The first-line treatment for PBC is ursodeoxycholic acid, but some patients do not respond well to it. Obeticholic acid is a second-line treatment option. Fenofibrate, predominantly PPAR-α agonist, and bezafibrate, a pan-PPAR agonist, are used in clinical practice as anticholestatic agents in order to improve serum biochemistry in PBC. Seladelpar, a peroxisome proliferator-activated receptor-delta (PPAR-δ) agonist, is a once-daily, oral, potent, and selective drug that has demonstrated potent anti-cholestatic effects in clinical studies. PPAR-δ is unique among PPAR isotypes, with broad expression in cells that play a key role in the pathobiology of primary biliary cholangitis: hepatocytes, cholangiocytes, Kupffer cells, and stellate cells (Odegaard et al., 2008; Iwaisako et al., 2012; Xia et al., 2012). The activation of PPAR-δ by seladelpar releases fibroblast growth factor 21 (FGF-21) from hepatocytes, which in turn reduces the accumulation of bile acids by inhibiting the expression of cholesterol 7α-hydroxylase, the rate-limiting enzyme for bile acid synthesis (Shukla and Misra, 2025). Studies show that seladelpar decreases proinflammatory macrophages, an effect that is consistent with the known effect of PPAR-δ to promote the anti-inflammatory M2 phenotype in Kupffer cells and macrophages (Odegaard et al., 2008; Bojic et al., 2014; Haczeyni et al., 2017).

## 5 Intrahepatic cholestasis of pregnancy

Intrahepatic cholestasis of pregnancy is a pregnancy-specific liver disorder that usually develops in the late second or third trimester. The condition is characterized by intense itching (pruritus), most commonly on the palms and soles, and by elevated maternal serum bile acid concentrations. Symptoms typically resolve spontaneously within days after delivery, which highlights the transient and pregnancy-dependent nature of the disease (Ozkan et al., 2015; European Association for the Study of the Liver, 2023). The exact cause of ICP is not fully understood, but current evidence points to a multifactorial pathogenesis. The disorder develops from the interaction between hormonal factors, genetic predisposition, and environmental influences. During pregnancy, high levels of estrogens and progesterone metabolites can impair bile secretion by reducing the activity of bile acid transport proteins in hepatocytes, the main functional cells of the liver. In genetically susceptible women, this hormonal effect is amplified by inherited variants in genes such as ABCB4 (encoding MDR3, a phospholipid transporter) and ABCB11 (encoding BSEP, the bile salt export pump) (Stättermayer et al., 2020; Xiao et al., 2021). During pregnancy, estrogens and sulfated progesterone metabolites can depress bile-acid transport by trans-inhibiting BSEP and dampening FXR signaling; they also interfere with NTCP-mediated uptake, together promoting intrahepatic retention of bile acids (Vallejo et al., 2006; Chen et al., 2015; Dumančić et al., 2024; Jasak et al., 2025). Accumulating hydrophobic bile acids then injure hepatocytes and cholangiocytes through oxidative/ER stress, mitochondrial dysfunction, and apoptosis; the biliary epithelium is particularly vulnerable when ABCB4/MDR3-dependent phosphatidylcholine secretion is reduced, because the loss of phospholipid “buffering” increases the detergent toxicity of bile within canaliculi and ducts (Wasmuth et al., 2007; Perez and Briz, 2009). The most significant and feared complication of ICP is the increased risk of intrauterine fetal death (stillbirth). The association between the severity of maternal cholestasis, quantified by serum total bile acid (TBA) concentrations, and the risk of stillbirth has been clearly delineated by large-scale meta-analyses (Geenes et al., 2014; Kawakita et al., 2015; Al-Obaidly et al., 2022; Huang et al., 2024; Zhou et al., 2024; Majewska et al., 2026). Critical findings from an individual patient data meta-analysis indicate that the risk of stillbirth in singleton pregnancies is significantly increased, specifically when maternal serum bile acid concentrations reach a threshold of ≥ 100 μmol/L (Sasaki et al., 2008). This finding is pivotal for clinical risk stratification. Importantly, the vast majority of women diagnosed with ICP have TBA levels persistently below this critical cutoff. For this group, the evidence suggests that the risk of stillbirth is comparable to that of the general obstetric population, provided that rigorous monitoring is implemented, including serial bile acid measurements throughout the remainder of the pregnancy to detect any potential increase towards the high-risk threshold (Ovadia et al., 2019). Collectively, these mechanisms explain bile duct injury and the ductular reaction seen in ICP, while guideline documents emphasize its clinical reversibility and the central role of bile-acid–focused evaluation and management.

## 6 Role of bile acids in pathogenesis

Bile acids, the principal components of bile, are involved in all stages of PBC pathogenesis. They influence immune cell activity, alter the intestinal microbiota, and activate hepatic stellate cells, thereby contributing to the initiation, progression, and perpetuation of the disease (Yang and Duan, 2016).

The initiation is the moment when a triggering factor starts an immune response against bile acids (Beuers et al., 2010). Progression, with cholestasis manifestation, is caused by the retention of bile acids inside the cells, which results in their apoptosis and consequently damages to the bile ducts and liver cells. These events activate immunological mechanisms and a local inflammatory response (Pablo Arab et al., 2017). The perpetuation stage is characterized by bile acid-induced liver fibrosis. The induction might be direct, through the activation of signaling pathways in stellate cells, or indirect, through hepatocytes apoptosis and tumor growth factor β (β-TGF) released by dead cells (Svegliati-Baroni et al., 2025).

Additionally, impaired bile flow promotes intrahepatic accumulation of bile acids and bilirubin, inducing endoplasmic reticulum stress, mitochondrial injury, and hepatocellular damage. These processes stimulate the release of pro-inflammatory cytokines and oxidative stress markers such as 8-hydroxy-2’-deoxyguanosine (8-OHdG), which reflects DNA damage in both biliary epithelial and hepatocyte nuclei (Copple et al., 2010; Ortiz et al., 2023).

Bile acids act as signaling molecules through nuclear and membrane receptors, including the farnesoid X receptor, pregnane X receptor (PXR), and Takeda G-protein receptor 5 (TGR5). Disruption of these signaling pathways in PSC contributes to impaired bile acid homeostasis, inflammation, and fibrosis (Fickert and Wagner, 2017). Clinical studies have also shown altered bile acid composition in PSC patients, with increased levels of hydrophobic and potentially hepatotoxic bile acids and reduced protective hydrophilic fractions, such as ursodeoxycholic acid. This imbalance may further perpetuate bile duct injury and contribute to the disease’s progressive fibro-inflammatory phenotype (Özdirik and Schnabl, 2024; Kayashima et al., 2024; Li et al., 2024).

In ICP, bile acids are both the signal and the toxin: pregnancy hormones and genetic susceptibility converge to impair hepatobiliary bile-acid handling, leading to intrahepatic accumulation of hydrophobic bile acids that damage hepatocytes and the cholangiocytes of bile-duct epithelium. Mechanistically, sulfated progesterone metabolites that rise during late gestation can antagonize the farnesoid X receptor—the master nuclear receptor that maintains bile-acid homeostasis—and trans-inhibit transporters that move bile acids across hepatocyte membranes (notably the bile salt export pump, BSEP/ABCB11, and the sodium-taurocholate cotransporting polypeptide, NTCP). This hormonal effect reduces bile-acid excretion and favors retention within the liver (Abu-Hayyeh et al., 2013). Consensus statements and recent reviews emphasize this hormone–gene–bile-acid triad as the core of ICP pathogenesis and guide clinical monitoring around maternal serum bile acids, which reflect the underlying transport failure and toxic load (Piechota and Jelski, 2020).

## 7 Impact of oxidative stress on hepatocytes and cholangiocytes

Biliary cholestasis is closely associated with inflammation, oxidative stress, and subsequent hepatocellular damage. Oxidative stress is a major contributor to post-translational modifications of cellular proteins, and the generation of reactive oxygen species (ROS) during chronic inflammation is considered central to the progression of chronic liver diseases (Shearn et al., 2018). In the liver, elevated levels of lipid peroxidation and accumulation of electrophilic α/β-unsaturated fatty acid derivatives, such as 4-hydroxynonenal (4-HNE), acrolein, and malondialdehyde (MDA), serve as key markers of oxidative stress. Although the exact source of reactive aldehydes in cholestasis remains unclear, bile duct ligation (BDL) experiments in mice have suggested that neutrophil infiltration contributes significantly to their formation (Shearn et al., 2019).

Clinical studies in patients with choledocholithiasis, neutrophilia, and hyperbilirubinemia showed a strong positive correlation with MDA levels, indicating lipid peroxidation as the final product of oxidative stress. In contrast, other inflammatory and biochemical markers of cholestasis did not correlate significantly. This suggests that neutrophil counts and bilirubin levels, both easily obtained in routine laboratory tests, may represent important parameters for estimating the extent of liver injury in biliary obstruction (Damnjanović et al., 2013).

At the cellular level, hydrophobic bile acids induce lipid peroxidation in macrophages. Kupffer cells (KCs), the liver-resident macrophages, not only play a central role in responding to injury but also act as potent ROS generators, primarily through NADPH oxidase (NOX2) activity in association with Toll-like receptor (TLR) signaling. Upon activation by pro-fibrogenic stimuli, such as alcohol or endotoxins, KCs release biologically active mediators—including chemokines, cytokines, adhesion molecules, and ROS—that promote hepatocyte and hepatic stellate cell (HSC) injury and fibrogenesis. A rabbit model of non-alcoholic steatohepatitis (NASH) further demonstrated that KCs are key contributors to lipid peroxide generation, leading to steatosis (Luangmonkong et al., 2018).

Elevated products of lipid peroxidation have also been reported in patients with PBC and PSC, with increased lipid peroxide levels corresponding to decreased serum antioxidant capacity (Shearn et al., 2018). Gene expression analyses in hepatocytes from cholestatic liver disease (CLD) patients revealed differential expression of multiple bile acid transporters and stress response genes. Notably, PPARα expression was significantly upregulated, suggesting an adaptive response aimed at enhancing bile acid detoxification and fatty acid β-oxidation during cholestasis (Ortiz et al., 2023). PPARα is also known to directly induce transcription of the multidrug resistance protein 3 (MDR3/ABCB4), thereby facilitating biliary phosphatidylcholine secretion, a protective mechanism against bile acid toxicity (Ghonem et al., 2014).

Oxidative DNA damage represents another hallmark of CLD. Immunostaining for 8-hydroxydeoxyguanosine (8-OHdG), a ROS-induced DNA lesion, demonstrated significantly higher fluorescence intensity in hepatocytes from CLD patients compared to controls, indicating enhanced nuclear and mitochondrial DNA damage (Nishio et al., 2019). The distribution of 8-OHdG primarily in the cytoplasm highlighted mitochondrial involvement in oxidative injury. Although variable across patient samples, likely reflecting different disease stages, the findings strongly suggest enhanced oxidative stress–driven DNA damage in CLD. Parallel analyses showed increased expression of NRF2 and GPX4—regulators of antioxidant responses—although these changes did not reach statistical significance, further supporting the notion of a reactive but insufficient adaptive response.

Autophagy also plays a critical role in cholestatic liver injury. In human PBC and PSC, autophagy is upregulated, as evidenced by increased expression of p62 and LC3 in biliary epithelial cells and periportal hepatocytes adjacent to damaged bile ducts. Experimental BDL models in mice confirmed that disruption of autophagy through deletion of Atg5 or Atg7 results in increased p62 accumulation and exacerbated intrahepatic cholestasis, highlighting the importance of functional autophagy in limiting injury (Gao et al., 2014; Kim et al., 2018). Moreover, (Shearn et al. (2022)). demonstrated that p62, an autophagy adaptor protein, is a target of reactive aldehydes in human and murine cholestatic liver disease, with accumulation observed in periportal hepatocytes and subsets of macrophages surrounding areas of inflammation and fibrosis (Shearn et al., 2022).

Collectively, these findings suggest that cholestasis-driven oxidative stress promotes lipid peroxidation, DNA damage, and inflammatory activation, while impaired autophagy amplifies tissue injury. The interplay between ROS production, immune cell activation, nuclear receptor responses such as PPARα, and defective autophagy provides a unifying framework for understanding hepatocellular and cholangiocellular injury in cholestatic and autoimmune liver diseases.

A study by (Dallio et al. (2024a)). involving 41 PBC patients demonstrated significantly elevated levels of derivatives of reactive oxygen metabolites (dROMs) and reduced biological antioxidant potential (BAP) compared to healthy controls. Moreover, the imbalance between high dROM and low BAP correlated with disease progression, particularly with increasing liver fibrosis and steatosis. Interestingly, no significant differences were observed between PBC-AMA+ and PBC-AMA– patients. dROM levels also showed associations with clinical and biochemical markers of disease progression, including alkaline phosphatase (ALP), aspartate aminotransferase (AST), gamma glutamyltransferase (GGT), and albumin. These relationships are likely linked to mitochondrial dysfunction, which plays a central role in PBC pathogenesis by promoting ROS generation and releasing mitochondrial AST isoenzymes. Additionally, elevated ALP secretion may be induced by oxidative processes occurring in vascular and bone tissues (Dallio et al., 2024a).

Superoxide dismutase (SOD) activity appears to increase during the early stages of PBC, possibly reflecting a compensatory mechanism to neutralize rising ROS levels. In contrast, at more advanced stages, SOD and other antioxidant defenses tend to stabilize or decline. This may be partially explained by the influence of dietary antioxidant intake, which provides some protection against oxidative damage but does not fully counterbalance ongoing oxidative stress (Kaffe et al., 2015).

Several studies confirm the role of oxidative stress in PBC progression. In a cohort of 274 untreated patients, 68% showed elevated markers of oxidative stress in liver biopsy samples, including anti-MDA–HSA IgG autoantibodies, reflecting lipid peroxidation (Sorrentino et al., 2010). Immunohistochemical analyses also revealed cytoplasmic localization of 8-OHdG in hepatocytes and biliary epithelial cells, highlighting mitochondrial and nuclear DNA damage caused by ROS (Copple et al., 2010; Ortiz et al., 2023).

Patients with PBC further exhibit dysregulation of antioxidant defense pathways. The transcription factor Nrf2, a master regulator of antioxidant responses, showed markedly altered activity: protein levels were 3.3-fold higher compared to controls, whereas mRNA expression was reduced 3.5-fold. Downstream targets of Nrf2, such as heme oxygenase-1 (HO-1), were significantly suppressed, with HO-1 mRNA reduced 9-fold and protein levels reduced 2-fold. Similarly, glutamate-cysteine ligase catalytic subunit (GCLC) expression was reduced 2.7-fold (Dallio et al., 2024a). These findings indicate impaired transcriptional regulation of antioxidant defenses despite compensatory increases in Nrf2 protein.

Epigenetic mechanisms further contribute to this imbalance. Increased expression of microRNAs miR-132 and miR-34a, together with upregulation of the adaptor protein p62 (which degrades Keap1), were observed in PBC livers. In parallel, reduced expression of antioxidant enzymes such as SOD1, SOD2, and GPx1 was detected in patients with liver fibrosis, suggesting that persistent oxidative imbalance accelerates disease progression (Dallio et al., 2024a).

Lipid peroxidation represents a critical mechanism of oxidative damage during cholestasis and autoimmune liver disease. Under conditions of oxidative stress, reactive oxygen species such as superoxide and hydrogen peroxide interact with membrane lipids, generating reactive aldehydes—most notably 4-hydroxy-2-nonenal (HNE) and MDA. These highly reactive metabolites form covalent adducts with proteins, resulting in their inactivation and acquisition of antigenic properties, which subsequently trigger immune-mediated degradation and contribute to the progression of fibrosis (Kawamura et al., 2000; Wójcik et al., 2021). Dysregulation between pro-oxidant lipid peroxidation products and antioxidant defenses disrupts cellular homeostasis, leading to membrane destabilization, impaired cellular function, and the development of autoantibody responses, including the generation of IgG-class autoantibodies as markers of oxidative injury (Yesilova et al., 2005; Dallio et al., 2024a).

Clinical and experimental studies confirm the pivotal role of lipid peroxidation in cholestatic liver diseases. In patients with PBC and PSC, levels of lipid peroxidation markers—including 8-isoprostane, MDA, 4-HNE, and an elevated GSSG/total GSH ratio—have been consistently elevated compared to healthy controls. In contrast, reduced levels of reduced glutathione (GSH) were observed, correlating with overexpression of perinuclear 4-HNE in damaged bile ducts of PBC patients. Notably, 8-isoprostane levels were elevated irrespective of cirrhosis, while the GSSG/total GSH ratio was significantly higher only in cirrhotic individuals. These findings suggest that oxidative imbalance emerges early in disease development and worsens with progression, supporting the hypothesis that lipid peroxidation is not merely a consequence but also a driver of disease progression. Early depletion of intrahepatic glutathione reserves may thus promote lipid peroxidation, stimulate collagen synthesis, and accelerate fibrogenesis (Kaffe et al., 2015).

Animal models further reinforce this concept. In the 1990s, (Muriel and Suarez (1994)). established a cholestatic model in Wistar rats using bile duct ligation (BDL). Markers of hepatocellular damage, including alkaline phosphatase (AP), γ-glutamyltransferase (γ-GT), bilirubin, and glutamate pyruvate transaminase (GPT), increased within 24 h of ligation. GPT normalized by day 8, likely reflecting the depletion of hepatocytes undergoing apoptosis, while other enzymes remained elevated. Lipid peroxidation, measured by MDA levels, increased markedly from day 3, suggesting that oxidative stress is both an early event and a consequence of hepatocyte apoptosis induced by biliary obstruction (Muriel and Suarez, 1994).

Histopathological investigations in PBC provide further insight into lipid peroxidation–associated injury. In biopsies from 20 PBC patients, HNE–protein conjugates were identified in all cases, not only in damaged but also in morphologically intact biliary epithelial cells (BECs). These conjugates localized predominantly to bile ducts smaller than 100 μm, which are specifically targeted in PBC, and were also observed in hepatocytes, particularly in patients with stage III disease and periportal cholestasis. The presence of HNE adducts in apparently healthy cells suggests that lipid peroxidation occurs early in the disease process and may contribute directly to bile duct injury rather than being solely secondary to inflammatory infiltration (Yesilova et al., 2005).

Complementary studies assessing urine and serum biomarkers in PBC patients demonstrated elevated 8-isoprostane and MDA levels, along with reduced total antioxidant capacity and decreased plasma glutathione concentrations, approximately 30% of normal controls. Disease stage, according to Ludwig’s classification, correlated with urinary 8-isoprostane and serum bile acid levels, highlighting the direct role of bile acids as promoters of oxidative damage, either through ROS generation or macrophage activation. These findings underscore the contribution of oxidative pathways from the earliest stages of PBC and suggest that lipid peroxidation is a key factor in driving fibrosis and cirrhosis (Kawamura et al., 2000).

Beyond classical oxidative stress markers, immune-regulatory molecules also reflect the interplay between oxidative damage and inflammation in PBC. For example, patients with high-risk disease exhibit elevated serum levels of CD44, a hyaluronic acid receptor that facilitates inflammatory cell recruitment. Experimental findings indicate that reduced CD44 expression leads to diminished pro-inflammatory cytokine synthesis, supporting its role in modulating immune-mediated inflammation in PBC (Tian et al., 2023).

Oxidative stress is increasingly recognized as a key pathogenic factor in primary sclerosing cholangitis. Clinical studies have demonstrated that patients with PSC exhibit elevated markers of oxidative damage, including derivatives of reactive oxygen metabolites (dROM) and malondialdehyde (MDA), along with reduced antioxidant capacity; these alterations correlate with disease severity and prognosis (Oyama et al., 2023). Mechanistic insights further strengthen this concept. (Shearn et al. (2019)). showed that cholestatic liver disease results in increased production of reactive aldehydes and an atypical periportal antioxidant response, implicating aldehyde-driven injury in PSC progression. More recently, (Shearn et al. (2022)). demonstrated that oxidative stress–derived aldehydes covalently modify the autophagic protein p62, leading to defective autophagy and exacerbation of cholestatic liver injury. In addition, experimental models such as Mdr2–/– mice, which recapitulate PSC-like pathology, confirm that accumulation of hydrophobic bile acids induces ROS production, causing hepatocyte and cholangiocyte injury and promoting periductal fibrosis (Shearn et al., 2022). Together, these findings provide consistent clinical and experimental evidence that oxidative stress is not merely a byproduct of cholestasis but a driving mechanism in the pathogenesis and progression of PSC.

Across ICP, redox imbalance is reflected by lipid-peroxidation products, antioxidant enzyme activities, low-molecular-weight antioxidant pools, and composite redox indices. In a prospective case–control study, maternal plasma 8-iso-prostaglandin F2α (8-iso-PGF2α) and glutathione peroxidase (GPx) were lower in ICP vs. controls, and 8-iso-PGF2α correlated inversely with total bile acids (TBA), suggesting antioxidant pathway failure/exhaustion rather than a simple rise in isoprostanes with higher cholestatic load (Hu et al., 2015). Complementing this, other cohorts report higher global oxidative tone: total antioxidant status, total oxidative status, and the oxidative stress index (OSI) were all increased in ICP, with elevated neopterin pointing to immune–redox crosstalk (Ozler et al., 2014).

Dynamic thiol/disulfide homeostasis also shifts toward oxidation (lower native/total thiols, higher disulfides), offering a practical serum readout of oxidative stress in ICP (Sanhal et al., 2018).

In pregnant women with ICP, bile acid accumulation leads to upregulation of the nuclear factor kappa B (NF-κB) protein. NF-κB mediates the inflammatory response by activating Th1 and Th2 lymphocytes and stimulating the secretion of proinflammatory cytokines, including IL-4, IL-6, IL-12, and tumor necrosis factor α (TNF-α). (Zhang et al. (2018)). demonstrated a correlation between NF-κB overexpression and elevated peroxisome proliferator-activated receptor γ (PPAR-γ) levels in women with ICP. PPAR-γ regulates the body’s inflammatory response. Its overexpression is secondary to NF-κB upregulation, as evidenced by rosiglitazone administration in rat models. This treatment resulted in decreased levels of NF-κB, inflammatory mediators, and reactive oxygen species (ROS). Conversely, the administration of GW9662, a PPAR-γ inhibitor, exacerbated the inflammatory state (Zhang et al., 2018).

On the other hand, as demonstrated by (Wu et al., 2016), high concentrations of hydrophobic bile acids in patients with ICP lead to the downregulation of peroxiredoxin-3 (PRDX3) synthesis in trophoblast cells, resulting in accelerated placental senescence and dysfunction. PRDX3 is a mitochondrial matrix enzyme that helps balance intracellular oxidative stress. The suppression of its synthesis leads to mitochondrial dysfunction, manifested by a loss of mitochondrial membrane potential, decreased intracellular ATP content, and reduced mitochondrial DNA (mtDNA) copy number and mitochondrial gene transcripts (MT-CO1, MT-ND1, MT-ND6). These changes are accompanied by intracellular ROS accumulation and exacerbated oxidative stress. Furthermore, the decreased level of PRDX3 and the consequent overproduction of ROS lead to cell cycle arrest and trophoblast cellular senescence via the activation of p38-mitogen-activated protein kinase (MAPK) and the induction of p21WAF1/CIP and p16INK4A factors, which act as cell cycle inhibitors (Wu et al., 2016).

Oxidative stress enzyme markers show heterogeneity across studies and clinical contexts. Serum superoxide dismutase (SOD) can be elevated in ICP, scales with TBA, and discriminates disease/severity—especially in women co-infected with hepatitis B virus—supporting its use as a prognostic adjunct (Wang et al., 2022). By contrast, another study (Hu et al., 2015) found no between-group differences for SOD and decreased GPx levels in ICP patients. Malondialdehyde (MDA), a terminal lipid-peroxidation product, is higher in maternal and cord erythrocytes in ICP and associates with fetal distress, linking oxidative injury to perinatal risk (Zhu et al., 2019). Depletion of antioxidant reserves is also documented: classic work shows low selenium with reduced GPx activity in ICP, and more recent translational data demonstrate lower coenzyme Q10 and vitamin E in women (and estrogen-cholestasis rat models), consistent with consumption of antioxidant defenses and supporting exploration of adjunct antioxidant strategies alongside ursodeoxycholic acid (Kauppila et al., 1987). Finally, experimental models of maternal cholestasis demonstrate placental and fetal oxidative damage that is ameliorated by ursodeoxycholic acid, providing mechanistic plausibility for the human biomarker findings (Sokol et al., 2001; Geenes and Williamson, 2009).

## 8 Therapeutic approach

Given the well-established role of oxidative stress in the pathogenesis and progression of cholestatic liver diseases, including PSC, PBC, and ICP, it is increasingly important to evaluate whether current therapeutic strategies also modulate oxidative pathways.

Ursodeoxycholic acid exerts a reproducible anti-oxidative effect in cholestatic liver injury. In hepatocytes, UDCA boosts intracellular glutathione by up-regulating γ-glutamylcysteine synthetase and increasing thiol-rich proteins (e.g., metallothionein), thereby enhancing redox buffering and resistance to oxidative injury (Mitsuyoshi et al., 1999; Okada et al., 2008; Li et al., 2020; Wang X. et al., 2024). *In vivo*, UDCA suppresses lipid peroxidation in chronic bile-duct-ligated (CBDL) rats, lowering hepatic malondialdehyde (MDA) and related peroxidation indices (Ljubuncic et al., 2000; Jüngst et al., 2008; Dong et al., 2026). In animal models of secondary biliary cirrhosis, UDCA preserves mitochondrial function and prevents formation of 4-hydroxynonenal (4-HNE)–protein adducts, consistent with mitigation of mitochondrial oxidative stress (Serviddio et al., 2004). Ursodeoxycholic acid also modulates antioxidant/oxidant enzymes, decreasing MDA and xanthine oxidase activity while increasing catalase (and reducing downstream apoptotic signals) in BDL rats (Sokolovic et al., 2013). Hepatocellular studies indicate that UDCA can induce GSH synthesis via the PI3K/Akt/NRF2 pathway, offering a transcriptional explanation for its redox benefits; comprehensive reviews summarize these cytoprotective, anti-apoptotic, and choleretic actions across cholestatic disease (Beuers, 2006; Arisawa et al., 2009).

The therapeutic strategy in PBC requires individualized planning based on patient characteristics, including age, gender, biochemical profile, histopathology, presence of disease-specific antibodies, and fibrosis markers. Such evaluation allows risk stratification into low- and high-risk groups, which is critical for selecting treatment pathways (Khan et al., 2025). Ursodeoxycholic acid is the first-line therapy in PBC, recommended at a dose of 13–15 mg/kg/day. It has an excellent safety profile, with rare adverse effects (Robles-Díaz et al., 2021). Its hepatoprotective mechanisms include modulation of γ-glutamylcysteine synthetase, leading to increased glutathione synthesis, stimulation of bicarbonate secretion in hepatocytes and cholangiocytes, alkalinization of bile, and protection of the bile duct epithelium from the toxic effects of hydrophobic bile acids (Kaffe et al., 2015; Erice et al., 2018). Although UDCA improves biochemical and clinical outcomes, approximately 30–40% of patients show an incomplete response and remain at high risk of progression to cirrhosis and liver failure (Tian et al., 2023; Guo et al., 2024).

In ICP, UDCA is likewise considered the first-line therapeutic option, primarily aimed at reducing maternal symptoms and improving biochemical cholestasis. Standard dosing ranges from 10–15 mg/kg/day, administered in divided doses, with titration up to ~21 mg/kg/day in women with persistent symptoms or elevated bile acids (Lee et al., 2021; European Association for the Study of the Liver, 2023). Ursodeoxycholic acid enriches the bile acid pool with a less toxic, hydrophilic component, reduces the concentration of harmful hydrophobic bile acids, and improves hepatocellular and cholangiocellular resistance to injury. Clinically, most women report a marked reduction in pruritus within 1–2 weeks of therapy, and biochemical improvement in serum bile acid concentrations and aminotransferases is typically seen after 2–3 weeks (Lee et al., 2021).

With respect to perinatal outcomes, large randomized controlled trials, including the PITCHES trial, demonstrated that UDCA improves maternal biochemical markers and itching but does not significantly reduce adverse fetal outcomes, such as stillbirth or preterm delivery (Chappell et al., 2019). However, pooled analyses and individual-patient meta-analyses suggest a possible reduction in the combined risk of stillbirth or preterm birth in some subgroups (Ovadia et al., 2021). Therefore, while UDCA is safe in pregnancy and remains the mainstay of treatment, the prevention of adverse fetal outcomes continues to rely primarily on close biochemical monitoring and timely delivery guided by peak maternal bile acid levels rather than UDCA therapy alone (European Association for the Study of the Liver, 2023).

Role of UDCA in PSC remains controversial. Early pilot studies suggested that UDCA might improve biochemical markers of cholestasis in PSC by enriching the bile acid pool with a more hydrophilic component, reducing the detergent activity of hydrophobic bile acids, and thereby attenuating cholangiocyte injury (Lindor, 1997). Indeed, short-term therapy was associated with reductions in serum alkaline phosphatase and transaminases, suggesting a hepatoprotective effect at the biochemical level. However, subsequent large randomized controlled trials showed that high-dose UDCA (28–30 mg/kg/day) did not confer clinical benefit and, in fact, was associated with increased rates of adverse outcomes, including death, liver transplantation, and development of colorectal neoplasia in PSC patients with concomitant inflammatory bowel disease (Lindor et al., 2009b). In another study, higher doses of UDCA (17–23 mg/kg/day) were associated with lower mortality, but the difference was not statistically significant. Additionally, the UDCA dosage used in the study did not lower ALP levels (Olsson et al., 2005). Meta-analyses and guideline statements from the American Association for the Study of Liver Diseases (AASLD) and the European Association for the Study of the Liver (EASL) now agree that UDCA should not be routinely used in PSC; however, low-to-moderate doses (13–15 mg/kg/day) may be considered in selected cases, particularly when patients demonstrate symptomatic improvement or biochemical response without adverse events (European Association for the Study of the Liver, 2022; Bowlus et al., 2023).

Mechanistically, UDCA may still provide insights into PSC pathogenesis. Reducing the hydrophobicity of the bile acid pool highlights the role of toxic bile acid accumulation in cholangiocyte injury. The partial biochemical improvements observed in some PSC patients treated with UDCA underscore that bile acid composition is central to PSC pathogenesis. Still, that modulation by UDCA alone is insufficient to stop fibrosis and disease progression. Thus, while UDCA remains the cornerstone in PBC, its place in PSC is primarily of historical and mechanistic interest, and current management focuses instead on surveillance, symptom control, and liver transplantation in advanced stages.

Obeticholic acid (6-ethyl-chenodeoxycholic acid), a potent farnesoid X receptor agonist, can modulate redox homeostasis through several mechanisms that ultimately lower reactive oxygen species (ROS) burden: repression of bile-acid synthesis (↓CYP7A1), enhancement of canalicular bile-acid efflux (↑BSEP/ABCB11), and crosstalk with antioxidant pathways (e.g., NRF2-responsive genes). Preclinical work shows that OCA and other FXR agonists reduce hepatic oxidative stress—for example, in diet/ethanol-induced injury, OCA (6-ECDCA) decreased oxidative stress alongside steatosis improvement in mice; similar anti-oxidative effects of FXR activation are summarized in alcoholic and metabolic liver disease models (Manley and Ding, 2015). In cholestatic settings, OCA therapy in bile duct–ligated (BDL) mice has been reported to attenuate liver injury and oxidative stress–linked phenotypes (including when combined with a ferroptosis inhibitor), and to lessen downstream neural consequences of cholestasis; together, these data support an anti-oxidative, cytoprotective signal downstream of FXR *in vivo* (Gee et al., 2023). Beyond the liver, OCA mitigates lipid peroxidation and ROS in kidney ischemia–reperfusion and toxic models, partly via NRF2-mediated induction of antioxidant genes, reinforcing a mechanistic link between FXR activation and redox control (Gai et al., 2016).

Obeticholic acid, as a semi-synthetic bile acid analogue of chenodeoxycholic acid, was approved by the FDA in 2016 as a second-line therapy in UDCA non-responders. It is a potent agonist of the farnesoid X receptor, the key nuclear receptor regulating bile acid synthesis, transport, and enterohepatic circulation. By activating FXR, OCA suppresses bile acid synthesis through inhibition of CYP7A1, enhances canalicular bile acid efflux via upregulation of BSEP (bile salt export pump), and modulates intestinal bile acid reabsorption, thereby reducing the overall toxic bile acid burden within the liver (Zhang et al., 2017; Zhang et al., 2019; Alvaro et al., 2025). Obeticholic acid activates the FXR, at doses of 5–10 mg/day, reducing bile acid synthesis, increasing their transport into bile ducts, and lowering intrahepatic accumulation by roughly one-third. OCA also exhibits anti-inflammatory and antifibrotic effects, while improving enterohepatic circulation (Guo et al., 2024; Khan et al., 2025). However, safety concerns have been raised, as reports from 2021 indicated increased risk of hepatic decompensation and liver failure in patients with advanced cirrhosis, making OCA contraindicated in decompensated disease and in compensated cirrhosis with portal hypertension (Barba Bernal et al., 2023). Response to treatment can be monitored by assessing bilirubin, transaminases, GGT, and ALP at 6–12 months. Current evidence suggests that OCA improves transplant-free survival and can be safely used in patients with compensated cirrhosis (Child-Pugh A) (Alvaro et al., 2025).

Clinical experience with OCA in PSC remains limited and preliminary. Unlike in primary biliary cholangitis, where OCA has regulatory approval as second-line therapy, randomized controlled trials in PSC are sparse. Small phase II studies and translational work have indicated that OCA can reduce serum bile acid levels and improve markers of cholestasis, but the magnitude of benefit is inconsistent, and there is no clear evidence that OCA alters long-term outcomes such as fibrosis progression, transplant-free survival, or cancer risk (Kowdley et al., 2020).

Mechanisms of OCA action provide a strong theoretical rationale for its potential use in cholestatic liver diseases, including ICP, where impaired bile acid transport and accumulation of hydrophobic bile acids drive hepatocellular and cholangiocellular injury (Pellicciari et al., 2002; Hirschfield et al., 2015). Despite this mechanistic plausibility, the safety of OCA during pregnancy remains unestablished. Human data are extremely limited, and existing reports are insufficient to define a risk profile (U.S. Food and Drug Administration, 2016). Regulatory bodies consequently recommend avoiding the use of OCA in pregnancy as a precautionary measure (European Medicines Agency, 2016). Although certain animal studies found no overt fetal malformations at lower doses, higher doses near maternal toxicity thresholds were associated with reduced fetal weight, increased resorptions, and fewer viable fetuses, highlighting potential fetal toxicity at elevated exposure levels (Drugs.com, 2023).

Fibrates, originally developed as hypolipidemic agents, act as peroxisome proliferator-activated receptor (PPAR) agonists with activity on PPAR-α, PPAR-γ, and PPAR-δ. By modulating transcription of genes involved in lipid metabolism, bile acid homeostasis, and inflammation, fibrates have gained attention as potential adjunctive therapies in cholestatic liver diseases. Their beneficial effects in primary biliary cholangitis are increasingly supported by clinical evidence, showing improvements in ALP and pruritus when combined with ursodeoxycholic acid.

In PSC, evidence is far more limited. Pilot studies and retrospective cohorts have suggested that bezafibrate and fenofibrate may reduce cholestatic markers such as ALP and γ-GT in a subset of patients, reflecting improved bile duct injury and cholestasis (Corpechot et al., 2018; Honda et al., 2019). Small trials also noted reductions in pruritus, a significant symptomatic burden in PSC, consistent with their antipruritic effect in PBC. Mechanistically, fibrates may exert benefit by enhancing bile acid detoxification, stimulating MDR3-mediated phospholipid secretion, and dampening oxidative stress and inflammatory pathways via PPAR activation (Ghonem et al., 2015). However, despite these biochemical improvements, there is no conclusive evidence that fibrates alter disease progression, transplant-free survival, or cancer risk in PSC. While human data are limited, case reports have described the off-label use of fibrates in pregnant patients with severe hypertriglyceridemia—cases complicated or coexisting with ICP—suggesting a favorable safety profile during at least the first trimester, although robust efficacy data are lacking. Limited data suggest fibrate use is safe during the first trimester of pregnancy (de Vries and Beuers, 2019). Most notably, a nationwide Korean cohort study of 756,877 pregnancies (2012–2021) examined the association between maternal fibrate exposure and congenital malformations. Among 260 pregnancies exposed to fibrates during the first trimester, the prevalence of congenital anomalies was 10.77% versus 9.68% in unexposed offspring, showing no significant increase in risk (OR 1.13; 95% CI 0.75–1.70). The authors concluded that fibrates do not appear to raise the risk of congenital malformations, although they advised caution with prolonged exposure (Kay et al., 2024).

## 9 Conclusions

Cholestatic and autoimmune liver diseases such as primary sclerosing cholangitis, primary biliary cholangitis, and intrahepatic cholestasis of pregnancy share a common pathogenic core defined by bile duct injury, dysregulated bile acid metabolism, and oxidative stress. Evidence from both clinical and experimental studies highlights that oxidative stress is not merely a secondary phenomenon but an active driver of hepatocellular and cholangiocellular injury, perpetuating inflammation, fibrosis, and ultimately disease progression. Key redox biomarkers, including malondialdehyde (MDA), 8-hydroxydeoxyguanosine (8-OHdG), and derivatives of reactive oxygen metabolites, consistently correlate with disease severity, prognosis, and response to therapy (**Table 1**).

**Table 1:** Summary of clinical and experimental findings on hepatocellular and cholangiocellular injury, inflammation, fibrosis, genetic basis and treatment response in primary sclerosing cholangitis, primary biliary cholangitis, and intrahepatic cholestasis of pregnancy.

Pathway:	Primary sclerosing cholangitis	Primary biliary cholangitis	Intrahepatic cholestasis in pregnancy
Bile accumulation	Altered bile acid composition with increased hydrophobic/hepatotoxic bile acids and reduced protective hydrophilic fractions perpetuates bile duct injury	Impaired bile flow promotes intrahepatic accumulation of bile acids and bilirubin; Bile acids influence immune cell activity and activate hepatic stellate cells.	Hormonal factors (estrogens, progesterone metabolites) and genetic variants impair hepatobiliary bile-acid handling, leading to intrahepatic accumulation of hydrophobic bile acids
References:	(Özdirik and Schnabl, 2024; Kayashima et al., 2024; Li et al., 2024)	(Copple et al., 2010; Yang and Duan, 2016; Petersen et al., 2018)	(Vallejo et al., 2006; Abu-Hayyeh et al., 2013; Chen et al., 2015; Stättermayer et al., 2020; Xiao et al., 2021; Dumančić et al., 2024; Jasak et al., 2025)
Oxidative stress	Elevated markers of oxidative damage (dROM, MDA) and reduced antioxidant capacity correlate with disease severity; Reactive aldehydes modify the autophagic protein p62, causing defective autophagy	Elevated lipid peroxidation markers (8-isoprostane, MDA, 4-HNE) and reduced GSH; High dROMs and low BAP correlate with progression; Dysregulated Nrf2 pathway	Shifted thiol/disulfide homeostasis toward oxidation, higher TAS, TOS, and OSI; Downregulation of PRDX3 and overexpression of NF-κB lead to ROS accumulation and mitochondrial dysfunction
References:	(Shearn et al., 2019; Shearn et al., 2022; Oyama et al., 2023)	(Kawamura et al., 2000; Sorrentino et al., 2010; Kaffe et al., 2015; Dallio et al., 2024a)	(Muriel and Suarez, 1994; Kawamura et al., 2000; Yesilova et al., 2005; Sorrentino et al., 2010; Gao et al., 2014; Ozler et al., 2014; Hu et al., 2015; Kaffe et al., 2015; Wu et al., 2016; Sanhal et al., 2018; Zhang et al., 2018; Wójcik et al., 2021; Shearn et al., 2022; Oyama et al., 2023; Tian et al., 2023)
Cell injury	Cholangiocyte senescence (an arrested, pro-inflammatory state) correlates with histologic severity; Biliary constituents trigger injury via N-Ras signaling	T lymphocyte–mediated destruction of small interlobular and septal bile ducts; Biliary epithelial cells exhibit premature senescence, apoptosis, and overexpression of p21WAF1/Cip1 and p16INK4.	Hydrophobic bile acids injure hepatocytes and cholangiocytes through oxidative/ER stress and apoptosis; ROS overproduction causes trophoblast cellular senescence via p38-MAPK activation
References:	(Tabibian et al., 2014; Cazzagon et al., 2021)	(Kumagi and Heathcote, 2008; Sasaki et al., 2008; Lindor et al., 2009a; Zhao et al., 2023)	(Wasmuth et al., 2007; Perez and Briz, 2009; Wu et al., 2016)
Fibrosis	Senescent cholangiocytes secrete profibrotic factors and recruit myofibroblasts, leading to concentric periductal “onion-skin” fibrosis and stricturing	Develops through the activation of myofibroblastic cells in the hepatic mesenchyme, progressing to cirrhosis; Early glutathione depletion promotes collagen synthesis	Typically a transient, self-limiting condition occurring in the third trimester; symptoms and biochemical abnormalities usually resolve spontaneously within days after delivery
References:	(Cazzagon et al., 2021; European Association for the Study of the Liver, 2022; Bowlus et al., 2023)	(Kumagi and Heathcote, 2008; Lindor et al., 2009a; Kaffe et al., 2015; Zhao et al., 2023)	(Ozkan et al., 2015; European Association for the Study of the Liver, 2023)
Genetic basis	Arises from an interaction of genetic susceptibility, disordered gut–liver crosstalk (dysbiosis), and maladaptive biliary epithelial responses	Arises in genetically predisposed individuals exposed to environmental triggers (viral/bacterial infections, xenobiotics) or immune dysregulation	Inherited variants in ABCB4 (MDR3) and ABCB11 (BSEP) genes amplify hormonal impairment of bile secretion in genetically susceptible women
References:	(Kevans et al., 2016; Sabino et al., 2016)	(Reshetnyak, 2015)	(Stättermayer et al., 2020; Xiao et al., 2021)
Treatment response	High-dose UDCA is not recommended due to adverse outcomes, but low-to-moderate doses may be considered; OCA and fibrates have limited/inconsistent evidence for altering long-term outcomes	UDCA is first-line therapy, but 30-40% have an incomplete response; OCA is used as second-line; Fibrates act as adjunctive therapy to improve ALP and pruritus	UDCA reduces maternal pruritus and improves biochemical markers but does not significantly reduce adverse fetal outcomes alone; OCA is unestablished/avoided in pregnancy; Fibrates may be safe in the first trimester
References:	(Lindor et al., 2009b; Corpechot et al., 2018; Honda et al., 2019; Kowdley et al., 2020; European Association for the Study of the Liver, 2022; Bowlus et al., 2023)	(Zhang et al., 2017; Erice et al., 2018; Zhang et al., 2019; Robles-Díaz et al., 2021; Tian et al., 2023; Guo et al., 2024; Alvaro et al., 2025; Khan et al., 2025)	(European Medicines Agency, 2016; U.S. Food and Drug Administration, 2016; Chappell et al., 2019; de Vries and Beuers, 2019; Lee et al., 2021; Ovadia et al., 2021; Drugs.com, 2023; European Association for the Study of the Liver, 2023; Kay et al., 2024)

4-HNE – 4-hydroxynonenal, ALP – alkaline phosphatase, BAP – biological antioxidant potential, dROM – derivatives of reactive oxygen metabolites, ER – endoplasmic reticulum, GSH – glutathione reduced, MDA – malondialdehyde, NF-κB – nuclear factor kappa B, OCA – obetichiolic acid, OSI – oxidative stress index, PRDX3 – peroxiredoxin-3, ROS – reactive oxygen species, TAC – total antioxidant capacity, TOS – total oxidative status, UDCA – urosodexycholic acid.

Therapeutically, current agents such as ursodeoxycholic acid remain central to management, not only for their choleretic properties but also for their demonstrated antioxidative and cytoprotective effects. Newer strategies, including obeticholic acid and fibrates, target nuclear receptor pathways (FXR, PPARs) and offer additional anti-inflammatory and redox-modulating potential, though safety and efficacy—particularly in pregnancy—require further investigation. Importantly, clinical trials continue to reveal that while these agents improve biochemical and symptomatic outcomes, their impact on long-term prognosis and survival remains heterogeneous, underscoring the need for combination and personalized approaches.

Future research should focus on integrating redox biology into therapeutic design, with emphasis on identifying patient subgroups most likely to benefit from targeted antioxidant modulation. Moreover, the development of reliable, non-invasive biomarkers of oxidative stress may facilitate earlier detection, stratification of risk, and monitoring of therapeutic responses. Collectively, a deeper mechanistic understanding of how bile acid toxicity and oxidative stress converge on bile duct pathology will be pivotal for advancing precision medicine in PSC, PBC, and ICP, ultimately improving patient outcomes across these challenging conditions.
